# Association of the variants and haplotypes in the DOCK7, PCSK9 and GALNT2 genes and the risk of hyperlipidaemia

**DOI:** 10.1111/jcmm.12713

**Published:** 2015-10-23

**Authors:** Tao Guo, Rui‐Xing Yin, Wei‐Xiong Lin, Wei Wang, Feng Huang, Shang‐Ling Pan

**Affiliations:** ^1^ Department of CardiologyInstitute of Cardiovascular DiseasesThe First Affiliated HospitalGuangxi Medical UniversityNanningGuangxiChina; ^2^ Department of Molecular GeneticsMedical Scientific Research CenterGuangxi Medical UniversityNanningGuangxiChina; ^3^ Department of PathophysiologySchool of Premedical SciencesGuangxi Medical UniversityNanningGuangxiChina

**Keywords:** hyperlipidaemia, dedicator of cytokinesis 7, pro‐protein convertase subtilisin/kexin type 9, polypeptide N‐acetylgalactosaminyltransferase 2, single nucleotide polymorphisms

## Abstract

Little is known about the association between the single nucleotide polymorphisms (SNPs) and haplotypes of the dedicator of cytokinesis 7 (*DOCK7*), pro‐protein convertase subtilisin/kexin type 9 (*PCSK9*) and polypeptide N‐acetylgalactosaminyltransferase 2 (*GALNT2*) and serum lipid traits in the Chinese populations. This study was to determine the association between nine SNPs in the three genes and their haplotypes and hypercholesterolaemia (HCH)/hypertriglyceridaemia (HTG), and to identify the possible gene–gene interactions among these SNPs. Genotyping was performed in 733 HCH and 540 HTG participants. The haplotype of C‐C‐G‐C‐T‐G‐C‐C‐G [in the order of *DOCK7* rs1168013 (G>C), rs10889332 (C>T); *PCSK9* rs615563 (G>A), rs7552841 (C>T), rs11206517 (T>G); and *GALNT2* rs1997947 (G>A), rs2760537 (C>T), rs4846913 (C>A) and rs11122316 (G>A) SNPs] was associated with increased risk of HCH and HTG. The haplotypes of C‐C‐G‐C‐T‐G‐C‐C‐A and G‐C‐G‐T‐T‐G‐T‐C‐G were associated with a reduced risk of HCH and HTG. The haplotypes of G‐C‐G‐C‐T‐G‐C‐C‐A and G‐C‐G‐C‐T‐G‐T‐C‐G were associated with increased risk of HCH. The haplotypes of C‐T‐G‐C‐T‐G‐C‐C‐G, G‐C‐A‐C‐T‐G‐C‐C‐G and G‐C‐G‐C‐T‐G‐C‐C‐A were associated with an increased risk of HTG. The haplotypes of G‐C‐G‐C‐T‐G‐T‐C‐A and G‐C‐G‐T‐T‐G‐T‐C‐G were associated with a reduced risk of HTG. In addition, possible inter‐locus interactions among the *DOCK7*,*PCSK9* and *GALNT2* SNPs were also noted. However, further functional studies of these genes are still required to clarify which SNPs are functional and how these genes actually affect the serum lipid levels.

## Introduction

Cardiovascular disease (CVD) is the major cause of premature death in both European 1 and American countries 2 and the rest of the world 3. It is an important cause of disability 4 and contributes substantially to the escalating costs of health care 5. Hyperlipidaemia—the risk factor for CVD 6 and related complications 7 leading to high morbidity and mortality 8. The 2013 American College of Cardiology/American Heart Association Guideline on the Treatment of Blood Cholesterol to Reduce Atherosclerotic Cardiovascular Risk in Adults represents a major shift from prior cholesterol management guidelines 9. The new guidelines introduce several major paradigm shifts, which include: aiming for atherosclerotic CVD risk reduction 10 as opposed to targeting low‐density lipoprotein cholesterol (LDL‐C) levels 11, and recommend an integrated approach to managing hyperlipidaemia to decrease atherosclerotic CVD risk 12. Although lipid modification was mainly focused on reducing the LDL‐C level in the past 13, lowering total cholesterol (TC) 14, triglyceride (TG) 15 and LDL‐C levels were found to be more beneficial than lowering LDL‐C alone 16. Although the risk for hyperlipidaemia has largely been attributed to adult lifestyle factors 17 such as poor nutrition 18, lack of exercise 19 and smoking 20, there is now strong evidence suggesting that predisposition to the development of hyperlipidaemia begins with heredity 21. It has been demonstrated that identifications of gene variants involved in hyperlipidaemia could provide a clue to search for novel pathogenesis and thereby new therapeutic or preventive methods for CVD.

Very large genome‐wide association studies (GWAS) of hyperlipidaemia have identified few novel loci that appear to influence lipid metabolism 22, 23, 24, including the *DOCK7* 25, *PCSK9* 26 and *GALNT2* 27 loci on chromosome 1. Assessment of the association between the *DOCK7*,* PCSK9* and *GALNT2* loci identified through GWAS 28, 29, 30 with the risk of hyperlipidaemia has become fundamental in the validation of these signals. *DOCK7* (gene ID: 85440, MedGen: CN189147, OMIM: 615859) is located on chromosome 1p31.3 (Exon count: 53) and encodes for DOCK7 protein. The protein encoded by this gene is a guanine nucleotide exchange factor (GEF) that plays a role in axon formation 31 and neuronal polarization 32. The encoded protein displays GEF activity towards RAC1 and RAC3 Rho small GTPases, but not towards CDC42 33. DOCK7 interaction with TACC3 controls interkinetic nuclear migration and the genesis of neurons from radial glial progenitor cells during cortical development 34. Several transcript variants encoding different isoforms have been found for this gene 35. *PCSK9* (gene ID: 255738, MedGen: C1863551, OMIM: 603776) is located on chromosome 1p32.3 (Exon count: 14). This gene encodes a member of the subtilisin‐like pro‐protein convertase family, which includes proteases that process protein and peptide precursors trafficking through regulated or constitutive branches of the secretory pathway 36. The encoded protein undergoes an autocatalytic processing event with its pro‐segment in the ER and is constitutively secreted as an inactive protease into the extracellular matrix and trans‐Golgi network 37. It is expressed in liver, intestine and kidney tissues and escorts specific receptors for lysosomal degradation. It plays a role in cholesterol and fatty acid metabolism 38. Mutations in this gene have been associated with autosomal dominant familial HCH 39. *GALNT2* (gene ID: 2590, OMIM: 602274) is located on chromosome 1q41‐q42 (Exon count: 20). This gene encodes a member of the glycosyltransferase 2 protein family. Members of this family initiate mucin‐type O‐glycosylation of peptides in the Golgi apparatus. The encoded protein may be involved in O‐linked glycosylation of the immunoglobulin A1 hinge region. This gene may influence TG levels, and may be involved in type 2 diabetes, as well as several types of cancer 40.

Although the association of some *DOCK7, PCSK9* and *GALNT2* SNPs and serum lipid levels has been reported in several previous studies, the association of the novel variants and their haplotypes and possible gene–gene interaction with the risk of hyperlipidaemia has never been detected previously. Therefore, this study was performed (*i*) to assess the association of the *DOCK7* (rs1168013 and rs10889332), *PCSK9* (rs615563, rs7552841 and rs1126517) and *GALNT2* (rs1997947, rs2760537, rs4846913 and rs11122316) SNPs and serum lipid levels in individuals with HCH/HTG; (*ii*) to evaluate the association of their haplotypes with the risk of HCH/HTG and (iii) to identify the possible gene–gene interactions among these variants in the Chinese population.

## Materials and methods

### Study populations

The participants were recruited from Dongxing City, Guangxi Zhuang Autonomous Region, People's Republic of China in 2012. A total of 1869 participants were randomly selected from our stratified, randomized samples 41. There were 999 hyperlipidaemic (TC > 5.17 mmol/l and/or TG > 1.70 mmol/l) and 870 normolipidaemic (TC ≤ 5.17 mmol/l and TG ≤ 1.70) individuals, aged 18–80 years. The age and gender distribution were matched between the two populations. The participants with a history of CVD including coronary artery disease and stroke, diabetes, chronic illness including cardiac, renal, thyroid problems and/or a history of taking lipid‐modulating medications such as statins or fibrates were excluded. Within the hyperlipidaemic population to assess the association of SNPs with risk of HCH and HTG separately, the hyperlipidaemic populations were subdivided into hypercholesterolaemic (TC > 5.17 mmol/l) and hypertriglyceridaemic (TG >1.70 mmol/l) groups. Informed consents were obtained from all the participants after they have received a full explanation of the study. The study was reviewed and approved by the Ethics Committee of the First Affiliated Hospital, Guangxi Medical University.

### Epidemiological survey and biochemical measurements

The epidemiological survey was carried out by using internationally standardized methods and following a common protocol 42. Information on demographics, socio‐economic status, lifestyle, past medical history and family disease history was collected by using standardized questionnaires. The intake of alcohol was quantified as the number of liangs (about 50 g) of rice wine, corn wine, rum, beer or liquor consumed during the preceding 12 months. Alcohol consumption was categorized into groups of grams of alcohol per day: 0 (non‐drinkers), ≤25 and >25. Smoking status was categorized into the groups of cigarettes per day: 0 (non‐smokers), ≤20 and >20. The methods of blood pressure, height, weight and waist circumference measurements have been described in the previous studies. Fasting venous blood samples were taken and the levels of serum TC, TG, HDL cholesterol (HDL‐C), and LDL‐C in the samples were directly determined by enzymatic methods with commercially available kits, Tcho‐1, TG‐LH (RANDOX Laboratories Ltd., Crumlin Co. Antrim, UK), Cholestest N HDL, and Cholestest LDL (Daiichi Pure Chemicals Co. Ltd., Tokyo, Japan) respectively. Serum apolipoprotein (Apo) A1 and ApoB levels were assessed by the immunoturbidimetric assay by using a commercial kit (RANDOX Laboratories Ltd.). All determinations were performed with an autoanalyzer (Hitachi Ltd., Tokyo, Japan). The normal values of serum TC, TG, HDL‐C, LDL‐C, ApoA1 and ApoB levels and the ratio of ApoA1 to ApoB in our Clinical Science Experiment Centre were 3.10–5.17, 0.56–1.70, 1.16–1.42, 2.70–3.10 mmol/l, 1.20–1.60, 0.80–1.05 g/l and 1.00–2.50 respectively 43.

### SNP selection and genotyping

We selected nine SNPs in the *DOCK7*,* PCSK9* and *GALNT2* with the following assumptions: (*i*) Tag SNPs, which were established by Haploview (version 4.2; Broad Institute of MIT and Harvard, Cambridge, Massachusetts, USA) or functional SNPs in functional areas of the gene fragments (http://www.ncbi.nlm.nih.gov/SNP/snp); (*ii*) a known minor allele frequency (MAF) higher than 1% in European ancestry from the Human Genome Project Database and (*iii*) the target SNP region should be adequately replicated by PCR, and the polymorphic site should have a commercially available restriction endonuclease enzyme cleavage site to be genotyped with the restriction fragment length polymorphism (RFLP).

Genomic DNA was isolated from peripheral blood leucocytes using the phenol–chloroform method 41. Genotyping of nine SNPs was performed by PCR and RFLP. The characteristics of each SNP and the details of each primer pair, annealing temperature, length of the PCR products and corresponding restriction enzyme used for genotyping are summarized in Tables S1 and S2. The PCR products of the samples (two samples of each genotype) were sequenced with an ABI Prism 3100 (Applied Biosystems, International Equipment Trading Ltd., Vernon Hills, IL, USA) in Shanghai Sangon Biological Engineering Technology & Services Co. Ltd., Shanghai China.

### Statistical analysis

The statistical analyses were performed with the statistical software package SPSS 19.0 (SPSS Inc., Chicago, IL, USA). The quantitative variables were presented as the mean ± S.D. for those, that are normally distributed, and the medians and interquartile ranges for TG, which is not normally distributed. General characteristics between the two groups were compared by the Student's unpaired *t*‐test. The allele frequency and genotype distribution, as well as haplotype frequency between the groups were analysed by the chi‐squared test; and the Hardy–Weinberg equilibrium was verified with the standard goodness‐of‐fit test. Pair‐wise linkage disequilibria and haplotype frequencies among the SNPs were analysed using Haploview (version 4.2; Broad Institute of MIT and Harvard). The association between the genotypes and serum lipid parameters was tested by ancova. Any variants associated with the serum lipid parameter at a value of *P* < 0.005 (corresponding to *P* < 0.05 after adjusting for 9 independent tests by the Bonferroni correction) were considered statistically significant. Unconditional logistic regression was used to assess the correlation between the risk of hyperlipidaemia and genotypes (*DOCK7* rs1168013: GG = 1, CG = 2, CC = 3; rs10889332: CC = 1, CT = 2, TT = 3; *PCSK9* rs615563: GG = 1, AG = 2, AA = 3; rs7552841: CC = 1, CT = 2, TT = 3; rs11206517: TT = 1, GT = 2, GG = 3; *GALNT2* rs1997947: GG = 1, AG = 2, AA = 3; rs2760537: CC = 1, CT = 2, TT = 3; rs4846913: CC = 1, AC = 2, AA = 3 and rs11122316: GG = 1, AG = 2, AA = 3). Age, sex, body mass index (BMI), smoking and alcohol consumption were adjusted for the statistical analysis. Two‐sided *P* < 0.05 was considered statistically significant.

The inter‐locus interaction was analysed by generalized multifactor dimensionality reduction (GMDR) method, using GMDR software. The cross‐validation consistency score provides the degree of consistency when the selected interaction is identified as the best model among all possibilities considered. The testing balanced accuracy provides the degree of interaction, which accurately predicts the case–control status with scores between 0.50 (indicating that the model predicts no better than the chance) and 1.00 (indicating perfect prediction). A sign test or a permutation test provides *P*‐value for predicting accuracy to measure the significance of an identified model. The best model is selected as the combination of marker with maximum cross‐validation consistency and minimum prediction error.

## Results

### Characteristics of the studied populations

Tables 1 and 2 compare the general characteristics and serum lipid levels between the HCH and non‐HCH populations and between the HTG and non‐HTG populations respectively. Both HCH and HTG individuals had significantly higher anthropometric parameters than their control individuals (*P* < 0.05–0.001). The age and gender distribution, height, pulse pressure and the % of participants who smoked and consumed alcohol were not different between both the HCH and HTG individuals (*P* > 0.05). There was no difference in the level of systolic blood pressure between the HTG and non‐HTG populations (*P* > 0.05).

**Table 1:** Anthropometric and metabolic characteristics between the hypercholesterolaemic and non‐hypercholesterolaemic individuals

Characteristics	Hypercholesterolaemia	Non‐hypercholesterolaemia	*t* (χ^2^)	*P*‐value
Number	733	1136		
Male/Female	388/345	594/542	0.074	0.785^a^
Age (years)	58.34 ± 12.88	57.52 ± 13.33	1.323	0.186^b^
Height (cm)	158.54 ± 7.36	158.46 ± 7.87	0.199	0.843^b^
Weight (kg)	58.58 ± 9.83	57.63 ± 9.43	2.064	0.038^b^
Body mass index (kg/m^2^)	23.25 ± 3.24	22.91 ± 3.13	2.246	0.025^b^
Waist circumference (cm)	79.50 ± 9.34	78.53 ± 8.92	2.245	0.025^b^
Systolic blood pressure (mmHg)	136.10 ± 16.30	130.23 ± 19.46	2.720	0.007^b^
Diastolic blood pressure (mmHg)	81.90 ± 10.67	80.05 ± 10.19	3.720	0.000^b^
Pulse pressure (mmHg)	54.20 ± 14.69	50.18 ± 15.30	1.942	0.052^b^
Cigarette smoking, *n* (%)				
Non‐smoker	579 (78.99)	905 (79.66)		
≤20 Cigarette smoking/day	37 (5.05)	53 (4.67)	0.185	0.912^a^
>20 Cigarette smoking/day	117 (15.96)	178 (15.67)		
Alcohol consumption, *n* (%)				
Non‐drinker	578 (78.85)	915 (80.55)		
≤25 g/day	51 (6.96)	67 (5.90)	1.081	0.582^a^
>25 g/day	104 (14.19)	154 (13.55)		
Blood glucose level (mmol/l)	7.00 ± 1.53	6.48 ± 1.35	7.585	0.000^b^
Total cholesterol (mmol/l)	5.88 ± 0.57	4.42 ± 0.52	57.057	0.000^b^
Triglyceride (mmol/l)	1.52 (1.20)	1.27 (1.04)	−8.957	0.000^c^
HDL cholesterol (mmol/l)	1.41 ± 0.35	1.85 ± 0.33	−27.423	0.000^b^
Low‐density lipoprotein cholesterol (mmol/l)	3.19 ± 0.34	2.74 ± 0.40	25.910	0.000^b^
Apolipoprotein (Apo) A1 (g/l)	1.17 ± 0.11	1.40 ± 0.22	−28.573	0.000^b^
ApoB (g/l)	1.18 ± 0.25	0.96 ± 0.20	20.111	0.000^b^
ApoA1/ApoB	1.05 ± 0.25	1.52 ± 0.43	−29.493	0.000^b^

a Comparison between the hypercholesterolaemic and non‐hypercholesterolaemic individuals by chi‐squared test.

b Comparison between the hypercholesterolaemic and non‐hypercholesterolaemic individuals by *t*‐test.

c Comparison between the hypercholesterolaemic and non‐hypercholesterolaemic individuals by non‐parametric test. The values of triglyceride were presented as median (interquartile range).

**Table 2:** Anthropometric and metabolic characteristics between the hypertriglyceridaemic and non‐hypertriglyceridaemic individuals

Characteristics	Hypertriglyceridaemia	Non‐hypertriglyceridaemia	*t* (χ^2^)	*P*‐value
Number	540	1329		
Male/Female	292/248	680/649	1.301	0.254^a^
Age (years)	57.02 ± 13.37	57.35 ± 13.27	−0.489	0.625^b^
Height (cm)	158.35 ± 7.92	157.61 ± 7.64	1.888	0.059^b^
Weight (kg)	61.23 ± 10.78	56.24 ± 8.88	9.518	0.000^b^
Body mass index (kg/m^2^)	24.34 ± 3.38	22.60 ± 2.99	10.389	0.000^b^
Waist circumference (cm)	83.16 ± 9.11	77.19 ± 8.52	13.076	0.000^b^
Systolic blood pressure (mmHg)	134.29 ± 20.03	131.81 ± 43.79	1.261	0.208^b^
Diastolic blood pressure (mmHg)	82.73 ± 10.66	79.98 ± 10.22	5.123	0.000^b^
Pulse pressure (mmHg)	51.56 ± 16.33	51.83 ± 11.79	−0.150	0.881^b^
Cigarette smoking, *n* (%)				
Non‐smoker	435 (80.56)	1101 (82.84)		
≤20 Cigarette smoking/day	25 (4.63)	65 (4.89)	2.220	0.330^a^
>20 Cigarette smoking/day	80 (14.81)	163 (12.27)		
Alcohol consumption, *n* (%)				
Non‐drinker	426 (78.89)	1089 (81.94)		
≤25 g/day	31 (5.74)	87 (6.55)	5.362	0.068^a^
>25 g/day	83 (15.37)	153 (11.51)		
Blood glucose level (mmol/l)	7.05 ± 1.65	6.53 ± 1.33	6.519	0.000^b^
Total cholesterol (mmol/lL)	5.29 ± 0.92	4.87 ± 0.85	9.516	0.000^b^
Triglyceride (mmol/l)	2.16 (1.88)	1.21 (1.01)	−33.931	0.000^c^
HDL cholesterol (mmol/l)	1.60 ± 0.50	1.84 ± 0.48	−9.787	0.000^b^
Low‐density lipoprotein cholesterol (mmol/l)	2.98 ± 0.44	2.77 ± 0.41	9.506	0.000^b^
Apolipoprotein (Apo) A1 (g/l)	1.27 ± 0.22	1.33 ± 0.21	−4.971	0.000^b^
ApoB (g/l)	1.15 ± 0.24	1.01 ± 0.23	11.831	0.000^b^
ApoA1/ApoB	1.16 ± 0.34	1.38 ± 0.38	−12.622	0.000^b^

a Comparison between the hypertriglyceridaemic and non‐hypertriglyceridaemic individuals by chi‐squared test.

b Comparison between the hypertriglyceridaemic and non‐hypertriglyceridaemic individuals by *t*‐test.

c Comparison between the hypertriglyceridaemic and non‐hypertriglyceridaemic individuals by non‐parametric test. The values of triglyceride were presented as median (interquartile range).

### Genotype and allele frequencies

Tables 3 and 4 describe the genotype and allele frequencies of the *DOCK7*,* PCSK9* and *GALNT2* SNPs. The genotype distribution of all nine SNPs agreed with Hardy–Weinberg equilibrium (*P* > 0.05 for all). The genotype frequency of the rs1168013, rs10889332, rs615563, rs7552841, rs1997947, rs2760537 and rs4846913 SNPs and the allele frequencies of the rs10889332, rs615563, rs7552841, rs1997947, rs2760537 and rs4846913 SNPs were significantly different between the HCH and non‐HCH populations (*P* < 0.05–0.01). On the other hand, the genotype and allele frequencies of the rs10889332, rs615563, rs7552841, rs11206517, rs1997947, rs2760537, rs4846913 and rs11122316 SNPs and the allele frequency of the rs10889332, rs615563, rs7552841, rs11206517, rs1997947, rs2760537, rs4846913 and rs11122316 SNPs were significantly different between the HTG and non‐HTG groups (*P* < 0.05–0.001).

**Table 3:** The association between the *DOCK7*,* PCSK9* and *GALNT2* polymorphisms with hypercholesterolaemia

SNP	Genotype	Hypercholesterolaemia			OR (95% CI)	*P*‐value
		Genotype distribution, *n* (%)				
		Cases (*n* = 733)	Controls (*n* = 1136)	*P*‐value		
*DOCK7* rs1168013G>C	GG	312 (42.56)	538 (47.36)		1.02 (0.88, 1.19)	0.792
	CG/CC	421 (57.44)	598 (52.64)	0.027		
	MAF	492 (33.56)	725 (31.91)	0.293		
	HWE(*P*)	0.056	0.122			
*DOCK7* rs10889332 C>T	CC	369 (50.34)	649 (57.13)		0.84 (0.72, 0.98)	0.030
	CT/TT	364 (49.66)	487 (42.87)	0.007		
	MAF	439 (29.95)	571 (25.13)	0.001		
	HWE(*P*)	0.103	0.053			
*PCSK9* rs615563G>A	GG	436 (59.48)	764 (67.25)		0.77 (0.66, 0.91)	0.001
	AG/AA	297 (40.52)	372 (32.75)	0.001		
	MAF	345 (23.53)	420 (18.49)	0.000		
	HWE(*P*)	0.129	0.071			
*PCSK9* rs7552841 C>T	CC	459 (62.62)	801 (70.51)		0.75 (0.64, 0.89)	0.001
	CT/TT	274 (37.38)	335 (29.41)	0.000		
	MAF	315 (21.49)	370 (16.29)	0.000		
	HWE(*P*)	0.117	0.289			
*PCSK9* rs11206517 T>G	TT	608 (82.95)	974 (85.74)		0.91 (0.72, 1.16)	0.442
	GT/GG	125 (17.05)	162 (14.26)	0.218		
	MAF	135 (9.21)	172 (7.57)	0.075		
	HWE(*P*)	0.095	0.139			
*GALNT2* rs1997947G>A	GG	453 (61.80)	738 (64.96)		0.90 (0.76, 1.06)	0.215
	AG/AA	280 (38.20)	398 (35.04)	0.034		
	MAF	316 (21.56)	429 (18.89)	0.046		
	HWE(*P*)	0.671	0.066			
*GALNT2* rs2760537 C>T	CC	294 (40.11)	516 (45.42)		0.85 (0.75, 0.98)	0.025
	CT/TT	439 (59.89)	620 (54.58)	0.031		
	MAF	550 (37.52)	755 (33.23)	0.007		
	HWE(*P*)	0.217	0.201			
*GALNT2* rs4846913 C>A	CC	454 (61.94)	764 (67.25)		0.83 (0.70, 0.97)	0.022
	AC/AA	279 (38.06)	372 (32.75)	0.047		
	MAF	319 (21.76)	418 (18.40)	0.012		
	HWE(*P*)	0.251	0.136			
*GALNT2* rs11122316G>A	GG	292 (39.84)	438 (38.56)		1.01 (0.88, 1.17)	0.855
	AG/AA	441 (60.16)	698 (61.44)	0.331		
	MAF	544 (37.11)	837 (36.84)	0.868		
	HWE(*P*)	0.744	0.053			

MAF: minor allele frequency; HWE: Hardy–Weinberg equilibrium; DOCK7: dedicator of cytokinesis 7; PCSK9: pro‐protein convertase subtilisin/kexin type 9; GALNT2: polypeptide N‐acetylgalactosaminyltransferase 2.

**Table 4:** The association between the *DOCK7*,* PCSK9* and *GALNT2* polymorphisms with hypertriglyceridaemia

SNP	Genotype	Hypertriglyceridaemia			OR (95% CI)	*P*‐value
		Genotype distribution, *n* (%)				
		Cases (*n* = 540)	Controls (*n* = 1329)	*P*‐value		
*DOCK7* rs1168013G>C	GG	244 (45.19)	605 (45.52)		0.98 (0.83, 1.16)	0.832
	CG/CC	296 (54.81)	724 (54.48)	0.416		
	MAF	361 (33.43)	857 (32.24)	0.484		
	HWE(*P*)	0.367	0.517			
*DOCK7* rs10889332 C>T	CC	283 (52.41)	735 (55.30)		1.09 (0.91, 1.29)	0.365
	CT/TT	257 (47.59)	594 (44.70)	0.012		
	MAF	311 (28.80)	675 (25.40)	0.032		
	HWE(*P*)	0.053	0.495			
*PCSK9* rs615563G>A	GG	303 (56.11)	897 (67.49)		0.65 (0.55, 0.78)	0.000
	AG/AA	237 (43.89)	432 (32.51)	0.000		
	MAF	279 (25.83)	486 (18.28)	0.000		
	HWE(*P*)	0.181	0.079			
*PCSK9* rs7552841 C>T	CC	312 (57.78)	951 (71.56)		0.57 (0.48, 0.68)	0.000
	CT/TT	228 (42.22)	378 (28.44)	0.000		
	MAF	270 (25.00)	409 (15.39)	0.000		
	HWE(*P*)	0.058	0.922			
*PCSK9* rs11206517 T>G	TT	426 (78.89)	1155 (86.91)		0.63 (0.49, 0.81)	0.000
	GT/GG	114 (21.11)	174 (13.09)	0.000		
	MAF	126 (11.67)	182 (6.85)	0.000		
	HWE(*P*)	0.052	0.447			
*GALNT2* rs1997947G>A	GG	326 (60.37)	924 (69.53)		0.73 (0.61, 0.87)	0.001
	AG/AA	214 (39.63)	405 (30.47)	0.000		
	MAF	248 (22.96)	440 (16.55)	0.000		
	HWE(*P*)	0.179	0.778			
*GALNT2* rs2760537 C>T	CC	215 (39.82)	558 (41.99)		0.90 (0.78, 1.05)	0.195
	CT/TT	325 (60.18)	771 (58.01)	0.026		
	MAF	414 (38.33)	928 (34.91)	0.048		
	HWE(*P*)	0.079	0.546			
*GALNT2* rs4846913 C>A	CC	348 (64.44)	964 (72.54)		0.70 (0.58, 0.85)	0.000
	AC/AA	192 (35.56)	365 (27.46)	0.002		
	MAF	218 (20.19)	405 (15.24)	0.000		
	HWE(*P*)	0.286	0.052			
*GALNT2* rs11122316G>A	GG	179 (33.15)	551 (41.46)		0.81 (0.70, 0.95)	0.008
	AG/AA	361 (66.85)	778 (58.54)	0.003		
	MAF	443 (41.02)	938 (35.29)	0.001		
	HWE(*P*)	0.115	0.508			

MAF: minor allele frequency; HWE: Hardy–Weinberg equilibrium; DOCK7: dedicator of cytokinesis 7; PCSK9: pro‐protein convertase subtilisin/kexin type 9; GALNT2: polypeptide N‐acetylgalactosaminyltransferase 2.

### Genotypes and serum lipid levels

Table 5 depicts the association between the genotypes and serum lipid levels in the hypercholesterolaemic and normocholesterolaemic populations. After the Bonferroni correction of *P*‐values, the levels of TC (rs10889332 and rs7552841), TG (rs10889332, rs7552841, rs11206517, rs1997947, rs4846913 and rs11122316), HDL‐C (rs1168013, rs11206517, rs1997947 and rs4846913), LDL‐C (rs7552841 and rs1997947), ApoA1 (rs10889332, rs1997947 and rs4846913), ApoB (rs1168013, rs10889332 and rs7552841) and the ratio of ApoA1 to ApoB (rs1168013, rs10889332 and rs7552841) in the hypercholesterolaemic participants were different between the three genotypes (*P* < 0.005–0.001), whereas the levels of TC (rs1997947 and rs2760537), TG (rs10889332, rs615563, rs7552841, rs1997947, rs4846913 and rs11122316), ApoB (rs615563, rs7552841, and rs1997947), and the ratio of ApoA1 to ApoB (rs4846913) in the normocholesterolaemic individuals were different between the three genotypes (*P* < 0.005–0.001). Table 6 depicts the association between the genotypes and serum lipid levels in the hypertriglyceridaemic and normotriglyceridaemic populations. The levels of TG (rs1168013, rs10889332 and rs7552841), ApoA1 (rs4846913) and the ratio of ApoA1 to ApoB (rs10889332) in the hypertriglyceridaemic population were different between the genotypes (*P* < 0.005–0.001); whereas the levels of TC (rs1088933, rs615563 and rs7552841), TG (rs10889332, rs615563, rs1997947, rs2760537, rs4846913 and rs11122316) and HDL‐C (rs1168013, rs615563, rs11206517, rs1997947 and rs4846913), LDL‐C (rs10889332 and rs7552841), ApoA1 (rs1997947 and rs4846913), ApoB (rs10889332, rs615563, rs7552841 and rs11206517) and the ratio of ApoA1 to ApoB (rs615563, rs7552841, rs11206517 and rs1997947) in the normotriglyceridaemic population were different between the genotypes (*P* < 0.005–0.001).

**Table 5:** Association between the genotypes of *DOCK7*,* PCSK9* and *GALNT2* SNPs and serum lipid levels in the hypercholesterolaemic and non‐hypercholesterolaemic individuals

Genotype	*n*	TC (mmol/l)	TG (mmol/l)	HDL‐C (mmol/l)	LDL‐C (mmol/l)	ApoA1 (g/l)	ApoB (g/l)	ApoA1/ApoB
*DOCK7* rs1168013G>C								
Hypercholesterolaemia								
GG	312	5.81 ± 0.46	1.49 (1.17)	1.45 ± 0.30	3.15 ± 0.44	1.18 ± 0.10	1.14 ± 0.27	1.10 ± 0.28
CG	350	5.87 ± 0.57	1.54 (1.22)	1.39 ± 0.34	3.17 ± 0.33	1.17 ± 0.12	1.21 ± 0.22	1.02 ± 0.21
CC	71	5.90 ± 0.59	1.62 (1.27)	1.29 ± 0.50	3.21 ± 0.31	1.17 ± 0.11	1.26 ± 0.25	0.98 ± 0.18
*F*		1.082	4.931	10.176	1.771	0.590	16.215	20.545
*P*		0.340	0.085	0.000	0.171	0.554	0.000	0.000
Non‐hypercholesterolaemia								
GG	538	4.40 ± 0.52	1.25 (0.98)	1.87 ± 0.32	2.72 ± 0.40	1.39 ± 0.23	0.95 ± 0.20	1.53 ± 0.45
CG	471	4.42 ± 0.53	1.29 (1.04)	1.86 ± 0.31	2.73 ± 0.40	1.40 ± 0.21	0.97 ± 0.20	1.52 ± 0.42
CC	127	4.46 ± 0.45	1.28 (1.10)	1.83 ± 0.34	2.77 ± 0.41	1.40 ± 0.20	0.98 ± 0.19	1.48 ± 0.39
*F*		0.007	5.199	1.534	2.778	0.480	1.697	0.498
*P*		0.993	0.074	0.216	0.063	0.619	0.184	0.608
*DOCK7* rs10889332 C>T								
Hypercholesterolaemia								
CC	369	5.83 ± 0.55	1.46 (1.15)	1.42 ± 0.34	3.16 ± 0.37	1.21 ± 0.12	1.16 ± 0.21	1.08 ± 0.28
CT	289	5.86 ± 0.57	1.58 (1.23)	1.41 ± 0.34	3.19 ± 0.31	1.17 ± 0.12	1.16 ± 0.27	1.04 ± 0.22
TT	75	6.14 ± 0.57	1.68 (1.24)	1.37 ± 0.40	3.27 ± 0.30	1.17 ± 0.11	1.35 ± 0.29	0.99 ± 0.22
*F*		12.601	11.753	1.573	4.331	6.421	18.531	7.434
*P*		0.000	0.003	0.208	0.014	0.001	0.000	0.001
Non‐hypercholesterolaemia								
CC	649	4.41 ± 0.55	1.21 (1.00)	1.87 ± 0.31	2.72 ± 0.36	1.40 ± 0.23	0.95 ± 0.19	1.53 ± 0.42
CT	403	4.42 ± 0.50	1.40 (1.10)	1.85 ± 0.32	2.72 ± 0.40	1.40 ± 0.20	0.97 ± 0.21	1.53 ± 0.45
TT	84	4.44 ± 0.49	1.76 (1.04)	1.71 ± 0.38	2.78 ± 0.42	1.34 ± 0.21	1.00 ± 0.19	1.39 ± 0.38
*F*		0.027	38.484	5.031	2.648	1.047	1.304	0.997
*P*		0.974	0.000	0.007	0.071	0.351	0.272	0.369
*PCSK9* rs615563G>A								
Hypercholesterolaemia								
GG	436	5.84 ± 0.56	1.48 (1.14)	1.46 ± 0.40	3.18 ± 0.35	1.20 ± 0.11	1.16 ± 0.24	1.09 ± 0.23
AG	249	5.91 ± 0.57	1.52 (1.21)	1.41 ± 0.35	3.19 ± 0.32	1.18 ± 0.11	1.19 ± 0.29	1.06 ± 0.25
AA	48	6.01 ± 0.65	1.53 (1.20)	1.40 ± 0.33	3.25 ± 0.33	1.17 ± 0.11	1.21 ± 0.26	1.04 ± 0.25
*F*		2.815	0.934	0.072	1.422	0.939	2.901	0.535
*P*		0.061	0.627	0.930	0.242	0.392	0.056	0.586
Non‐hypercholesterolaemia								
GG	764	4.40 ± 0.51	1.23 (1.00)	1.89 ± 0.29	2.66 ± 0.31	1.42 ± 0.19	0.95 ± 0.19	1.54 ± 0.43
AG	324	4.45 ± 0.53	1.44 (0.98)	1.85 ± 0.34	2.73 ± 0.40	1.41 ± 0.22	0.99 ± 0.21	1.48 ± 0.40
AA	48	4.45 ± 0.56	1.47 (1.14)	1.84 ± 0.31	2.77 ± 0.42	1.37 ± 0.22	1.01 ± 0.26	1.47 ± 0.43
*F*		1.224	38.747	0.829	2.337	3.189	5.663	4.756
*P*		0.294	0.000	0.437	0.097	0.042	0.004	0.009
*PCSK9* rs7552841 C>T								
Hypercholesterolaemia								
CC	459	5.81 ± 0.52	1.49 (1.16)	1.41 ± 0.36	3.15 ± 0.34	1.20 ± 0.11	1.14 ± 0.23	1.07 ± 0.25
CT	233	5.91 ± 0.57	1.52 (1.25)	1.41 ± 0.33	3.22 ± 0.29	1.18 ± 0.12	1.23 ± 0.25	1.03 ± 0.26
TT	41	6.36 ± 0.84	1.84 (1.48)	1.39 ± 0.48	3.42 ± 0.43	1.17 ± 0.11	1.36 ± 0.33	0.94 ± 0.19
*F*		15.668	15.968	0.059	11.927	1.391	20.035	6.923
*P*		0.000	0.000	0.943	0.000	0.249	0.000	0.001
Non‐hypercholesterolaemia								
CC	801	4.41 ± 0.53	1.25 (1.00)	1.86 ± 0.33	2.73 ± 0.41	1.40 ± 0.22	0.94 ± 0.19	1.55 ± 0.44
CT	300	4.44 ± 0.50	1.32 (1.11)	1.82 ± 0.33	2.76 ± 0.40	1.39 ± 0.24	0.99 ± 0.20	1.46 ± 0.40
TT	35	4.51 ± 0.44	1.79 (1.21)	1.76 ± 0.28	2.76 ± 0.36	1.39 ± 0.17	1.08 ± 0.24	1.34 ± 0.39
*F*		0.792	36.882	1.762	0.688	0.082	10.172	4.915
*P*		0.453	0.000	0.172	0.503	0.922	0.000	0.007
*PCSK9* rs11206517 T>G								
Hypercholesterolaemia								
TT	608	5.86 ± 0.57	1.49 (1.17)	1.42 ± 0.34	3.19 ± 0.34	1.19 ± 0.10	1.17 ± 0.24	1.15 ± 0.41
GT	115	5.96 ± 0.58	1.65 (1.26)	1.40 ± 0.30	3.19 ± 0.32	1.18 ± 0.12	1.21 ± 0.27	1.06 ± 0.25
GG	10	6.00 ± 0.60	3.46 (2.08)	0.87 ± 0.60	3.32 ± 0.15	1.17 ± 0.12	1.26 ± 0.27	1.02 ± 0.22
*F*		1.649	17.220	12.269	0.896	0.562	5.430	1.955
*P*		0.193	0.000	0.000	0.409	0.570	0.005	0.142
Non‐hypercholesterolaemia								
TT	974	4.41 ± 0.52	1.26 (1.02)	1.85 ± 0.33	2.70 ± 0.23	1.40 ± 0.22	0.95 ± 0.19	1.54 ± 0.43
GT	152	4.42 ± 0.55	1.32 (1.09)	1.85 ± 0.30	2.74 ± 0.41	1.38 ± 0.21	1.01 ± 0.22	1.42 ± 0.45
GG	10	4.48 ± 0.50	1.39 (1.26)	1.62 ± 0.44	2.77 ± 0.40	1.29 ± 0.22	1.08 ± 0.19	1.19 ± 0.19
*F*		0.348	8.124	2.420	0.347	2.002	3.864	4.824
*P*		0.706	0.017	0.089	0.707	0.136	0.021	0.008
*GALNT2* rs1997947G>A								
Hypercholesterolaemia								
GG	453	5.79 ± 0.45	1.45 (1.15)	1.44 ± 0.33	3.00 ± 0.33	1.19 ± 0.11	1.18 ± 0.18	1.07 ± 0.26
AG	244	5.87 ± 0.53	1.65 (1.27)	1.39 ± 0.35	3.20 ± 0.31	1.17 ± 0.11	1.18 ± 0.25	1.04 ± 0.23
AA	36	5.91 ± 0.65	1.88 (1.51)	1.16 ± 0.42	3.20 ± 0.33	1.09 ± 0.10	1.19 ± 0.25	0.94 ± 0.18
*F*		0.935	35.099	8.223	6.491	11.573	0.482	2.612
*P*		0.393	0.000	0.000	0.002	0.000	0.618	0.074
Non‐hypercholesterolaemia								
GG	738	4.38 ± 0.52	1.23 (0.99)	1.85 ± 0.33	2.74 ± 0.42	1.41 ± 0.20	0.94 ± 0.18	1.55 ± 0.40
AG	367	4.48 ± 0.52	1.37 (1.13)	1.85 ± 0.31	2.74 ± 0.38	1.38 ± 0.22	0.99 ± 0.22	1.47 ± 0.47
AA	31	4.68 ± 0.38	2.04 (1.59)	1.73 ± 0.26	2.81 ± 0.29	1.35 ± 0.45	1.09 ± 0.18	1.28 ± 0.55
*F*		6.859	66.840	0.984	0.714	0.841	8.167	3.986
*P*		0.001	0.000	0.374	0.490	0.432	0.000	0.019
*GALNT2* rs2760537 C>T								
Hypercholesterolaemia								
CC	294	5.83 ± 0.61	1.49 (1.20)	1.42 ± 0.36	3.17 ± 0.44	1.18 ± 0.11	1.16 ± 0.25	1.08 ± 0.26
CT	328	5.90 ± 0.55	1.53 (1.17)	1.41 ± 0.32	3.19 ± 0.31	1.17 ± 0.12	1.19 ± 0.23	1.04 ± 0.23
TT	111	5.92 ± 0.52	1.60 (1.23)	1.36 ± 0.38	3.20 ± 0.32	1.17 ± 0.12	1.23 ± 0.28	1.02 ± 0.25
*F*		2.488	3.908	2.069	0.136	0.326	2.615	2.869
*P*		0.084	0.142	0.127	0.873	0.722	0.074	0.057
Non‐hypercholesterolaemia								
CC	516	4.37 ± 0.54	1.25 (1.00)	1.86 ± 0.32	2.71 ± 0.38	1.41 ± 0.22	0.94 ± 0.20	1.53 ± 0.46
CT	485	4.38 ± 0.54	1.26 (1.04)	1.84 ± 0.33	2.73 ± 0.42	1.40 ± 0.23	0.96 ± 0.20	1.52 ± 0.41
TT	135	4.47 ± 0.48	1.40 (1.19)	1.84 ± 0.35	2.76 ± 0.40	1.37 ± 0.18	0.97 ± 0.20	1.51 ± 0.45
*F*		6.233	15.432	1.249	2.153	1.248	3.367	0.741
*P*		0.002	0.000	0.287	0.117	0.287	0.035	0.477
*GALNT2* rs4846913 C>A								
Hypercholesterolaemia								
CC	454	5.87 ± 0.60	1.41 (1.14)	1.46 ± 0.30	3.15 ± 0.26	1.19 ± 0.11	1.18 ± 0.26	1.07 ± 0.25
AC	239	5.87 ± 0.52	1.68 (1.31)	1.34 ± 0.42	3.19 ± 0.33	1.15 ± 0.11	1.18 ± 0.24	1.02 ± 0.25
AA	40	6.00 ± 0.50	1.76 (1.51)	1.33 ± 0.40	3.20 ± 0.30	1.13 ± 0.12	1.21 ± 0.26	0.99 ± 0.24
*F*		1.098	54.273	8.734	0.222	7.807	0.114	2.669
*P*		0.334	0.000	0.000	0.801	0.000	0.892	0.070
Non‐hypercholesterolaemia								
CC	764	4.40 ± 0.52	1.22 (0.98)	1.86 ± 0.32	2.73 ± 0.41	1.42 ± 0.21	0.95 ± 0.20	1.56 ± 0.43
AC	326	4.44 ± 0.56	1.39 (1.13)	1.83 ± 0.34	2.76 ± 0.39	1.36 ± 0.21	0.98 ± 0.20	1.45 ± 0.42
AA	46	4.47 ± 0.51	1.92 (1.52)	1.75 ± 0.30	2.78 ± 0.37	1.36 ± 0.42	1.02 ± 0.14	1.34 ± 0.45
*F*		2.685	97.461	2.170	1.203	3.859	4.428	8.032
*P*		0.069	0.000	0.115	0.301	0.021	0.012	0.000
*GALNT2* rs11122316G>A								
Hypercholesterolaemia								
GG	292	5.80 ± 0.58	1.40 (1.10)	1.44 ± 0.31	3.17 ± 0.35	1.19 ± 0.10	1.15 ± 0.23	1.09 ± 0.27
AG	338	5.85 ± 0.53	1.50 (1.24)	1.42 ± 0.35	3.18 ± 0.31	1.18 ± 0.12	1.18 ± 0.24	1.06 ± 0.26
AA	103	5.93 ± 0.60	1.58 (1.29)	1.39 ± 0.36	3.21 ± 0.33	1.16 ± 0.11	1.19 ± 0.26	1.04 ± 0.23
*F*		2.240	26.385	0.745	1.271	2.462	0.701	1.505
*P*		0.107	0.000	0.475	0.281	0.086	0.496	0.223
Non‐hypercholesterolaemia								
GG	438	4.42 ± 0.50	1.24 (0.97)	1.88 ± 0.31	2.73 ± 0.41	1.40 ± 0.20	0.95 ± 0.19	1.54 ± 0.43
AG	559	4.41 ± 0.54	1.27 (1.04)	1.85 ± 0.32	2.73 ± 0.39	1.40 ± 0.27	0.96 ± 0.20	1.51 ± 0.43
AA	139	4.44 ± 0.50	1.40 (1.17)	1.83 ± 0.34	2.83 ± 0.42	1.39 ± 0.22	0.98 ± 0.21	1.50 ± 0.43
*F*		0.407	19.224	1.636	3.289	0.078	1.168	0.760
*P*		0.666	0.000	0.195	0.038	0.925	0.311	0.468

The values of TG were presented as median (interquartile range). The difference among the genotypes was determined by the Kruskal–Wallis test or the Wilcoxon‐Mann–Whitney test.

TC: total cholesterol; TG: triglyceride; HDL‐C: high ‐density lipoprotein cholesterol; LDL‐C: low‐density lipoprotein cholesterol; ApoA1: apolipoprotein A1; ApoB: apolipoprotein B; ApoA1/A poB: the ratio of a polipoprote in A1 to apolipoprote in B.

**Table 6:** Association between the genotypes of *DOCK7*,* PCSK9* and *GALNT2* SNPs and serum lipid levels in the hypertriglyceridaemic and non‐hypertriglyceridaemic individuals

Genotype	*n*	TC (mmol/l)	TG (mmol/l)	HDL‐C (mmol/l)	LDL‐C (mmol/l)	ApoA1 (g/l)	ApoB (g/l)	ApoA1/ApoB
*DOCK7* rs1168013G>C								
Hypertriglyceridaemia								
GG	244	5.13 ± 0.84	2.05 (1.85)	1.65 ± 0.56	2.85 ± 0.47	1.29 ± 0.23	1.13 ± 0.25	1.20 ± 0.38
CG	231	5.30 ± 0.95	2.19 (1.87)	1.58 ± 0.46	2.99 ± 0.42	1.27 ± 0.21	1.16 ± 0.23	1.13 ± 0.31
CC	65	5.33 ± 0.91	2.36 (2.01)	1.53 ± 0.50	3.00 ± 0.44	1.25 ± 0.20	1.17 ± 0.24	1.12 ± 0.22
*F*		0.976	12.551	3.153	2.837	3.954	1.009	5.229
*P*		0.378	0.002	0.044	0.059	0.020	0.365	0.005
Non‐hypertriglyceridaemia								
GG	605	4.83 ± 0.85	1.19 (0.98)	1.89 ± 0.47	2.76 ± 0.41	1.33 ± 0.23	1.00 ± 0.23	1.39 ± 0.38
CG	591	4.86 ± 0.75	1.20 (1.02)	1.82 ± 0.43	2.77 ± 0.41	1.32 ± 0.20	1.00 ± 0.23	1.38 ± 0.39
CC	133	4.91 ± 0.88	1.23 (1.08)	1.80 ± 0.49	2.79 ± 0.42	1.32 ± 0.19	1.04 ± 0.25	1.33 ± 0.35
*F*		1.595	4.320	5.876	1.407	0.972	0.960	0.865
*P*		0.203	0.115	0.003	0.245	0.379	0.383	0.421
*DOCK7* rs10889332 C>T								
Hypertriglyceridaemia								
CC	283	5.13 ± 0.92	2.06 (1.84)	1.64 ± 0.49	2.92 ± 0.48	1.28 ± 0.22	1.13 ± 0.26	1.20 ± 0.39
CT	203	5.18 ± 0.93	2.07 (1.88)	1.59 ± 0.53	2.94 ± 0.47	1.27 ± 0.21	1.15 ± 0.21	1.14 ± 0.30
TT	54	5.40 ± 0.90	2.93 (2.45)	1.45 ± 0.35	3.03 ± 0.40	1.26 ± 0.23	1.21 ± 0.28	1.08 ± 0.29
*F*		4.002	44.919	2.653	3.291	1.443	3.744	6.342
*P*		0.019	0.000	0.073	0.038	0.237	0.024	0.002
Non‐hypertriglyceridaemia								
CC	735	4.76 ± 0.79	1.16 (0.97)	1.86 ± 0.45	2.73 ± 0.40	1.36 ± 0.20	0.98 ± 0.21	1.39 ± 0.36
CT	513	4.95 ± 0.87	1.18 (0.99)	1.83 ± 0.47	2.81 ± 0.43	1.34 ± 0.23	1.02 ± 0.25	1.38 ± 0.41
TT	81	5.31 ± 1.03	1.26 (1.04)	1.83 ± 0.66	2.91 ± 0.42	1.31 ± 0.20	1.11 ± 0.32	1.30 ± 0.33
*F*		17.852	20.403	0.439	9.010	3.063	11.291	1.309
*P*		0.000	0.000	0.645	0.000	0.047	0.000	0.270
*PCSK9* rs615563G>A								
Hypertriglyceridaemia								
GG	303	5.25 ± 0.89	2.09 (1.84)	1.63 ± 0.39	2.97 ± 0.46	1.33 ± 0.21	1.13 ± 0.23	1.17 ± 0.38
AG	195	5.29 ± 0.93	2.19 (1.91)	1.60 ± 0.45	2.98 ± 0.41	1.28 ± 0.24	1.15 ± 0.24	1.16 ± 0.32
AA	42	5.50 ± 0.95	2.34 (1.93)	1.60 ± 0.54	3.06 ± 0.39	1.26 ± 0.20	1.25 ± 0.27	1.11 ± 0.26
*F*		1.734	1.193	0.164	0.954	3.026	2.706	0.161
*P*		0.178	0.551	0.849	0.386	0.049	0.068	0.851
Non‐hypertriglyceridaemia								
GG	897	4.80 ± 0.82	1.12 (0.97)	2.08 ± 0.59	2.75 ± 0.40	1.35 ± 0.23	0.98 ± 0.25	1.43 ± 0.33
AG	378	5.00 ± 0.90	1.18 (1.00)	1.84 ± 0.46	2.81 ± 0.44	1.33 ± 0.22	0.99 ± 0.22	1.41 ± 0.39
AA	54	5.02 ± 0.97	1.27 (1.06)	1.83 ± 0.47	2.88 ± 0.46	1.31 ± 0.20	1.05 ± 0.26	1.32 ± 0.36
*F*		6.248	24.916	5.777	3.104	0.818	7.986	6.656
*P*		0.002	0.000	0.003	0.045	0.441	0.000	0.001
*PCSK9* rs7552841 C>T								
Hypertriglyceridaemia								
CC	312	5.24 ± 0.92	2.05 (1.83)	1.69 ± 0.52	2.96 ± 0.42	1.32 ± 0.25	1.13 ± 0.22	1.17 ± 0.33
CT	186	5.28 ± 0.87	2.27 (1.98)	1.62 ± 0.49	2.98 ± 0.44	1.27 ± 0.21	1.15 ± 0.24	1.15 ± 0.35
TT	42	5.59 ± 1.24	2.47 (1.92)	1.56 ± 0.51	3.14 ± 0.54	1.25 ± 0.21	1.26 ± 0.33	1.11 ± 0.34
*F*		1.105	25.328	1.724	2.066	1.507	4.492	0.744
*P*		0.332	0.000	0.179	0.128	0.223	0.012	0.476
Non‐hypertriglyceridaemia								
CC	951	4.80 ± 0.82	1.19 (0.99)	1.96 ± 0.60	2.75 ± 0.41	1.37 ± 0.21	0.98 ± 0.22	1.41 ± 0.38
CT	347	5.00 ± 0.88	1.21 (1.06)	1.86 ± 0.48	2.82 ± 0.41	1.34 ± 0.24	1.06 ± 0.26	1.33 ± 0.37
TT	31	5.40 ± 1.08	1.24 (1.03)	1.83 ± 0.47	3.00 ± 0.52	1.32 ± 0.21	1.18 ± 0.30	1.22 ± 0.27
*F*		13.413	6.689	2.119	9.591	4.559	26.139	7.885
*P*		0.000	0.035	0.121	0.000	0.011	0.000	0.000
*PCSK9* rs11206517 T>G								
Hypertriglyceridaemia								
TT	426	5.23 ± 0.99	2.06 (1.84)	1.62 ± 0.52	2.97 ± 0.43	1.28 ± 0.25	1.14 ± 0.22	1.22 ± 0.53
GT	102	5.30 ± 0.90	2.17 (1.88)	1.57 ± 0.38	2.97 ± 0.48	1.27 ± 0.21	1.14 ± 0.22	1.16 ± 0.32
GG	12	5.52 ± 1.05	2.95 (2.28)	1.36 ± 0.33	3.07 ± 0.52	1.25 ± 0.26	1.19 ± 0.29	1.14 ± 0.38
*F*		0.250	7.802	1.673	0.141	0.490	1.418	0.279
*P*		0.779	0.020	0.189	0.869	0.613	0.243	0.757
Non‐hypertriglyceridaemia								
TT	1155	4.76 ± 0.73	1.20 (1.00)	1.85 ± 0.47	2.77 ± 0.42	1.34 ± 0.17	1.00 ± 0.23	1.40 ± 0.38
GT	166	4.84 ± 0.85	1.20 (1.01)	1.80 ± 0.44	2.81 ± 0.35	1.33 ± 0.22	1.07 ± 0.24	1.29 ± 0.37
GG	8	5.05 ± 0.86	1.26 (1.25)	1.74 ± 0.44	2.84 ± 0.31	1.32 ± 0.20	1.15 ± 0.27	1.20 ± 0.19
*F*		3.933	2.948	7.583	0.797	0.643	8.057	7.547
*P*		0.020	0.229	0.001	0.451	0.526	0.000	0.001
*GALNT2* rs1997947G>A								
Hypertriglyceridaemia								
GG	326	4.99 ± 0.71	2.12 (1.85)	1.65 ± 0.52	2.76 ± 0.54	1.28 ± 0.20	1.14 ± 0.23	1.17 ± 0.34
AG	180	5.25 ± 0.86	2.13 (1.90)	1.60 ± 0.49	2.99 ± 0.40	1.28 ± 0.24	1.14 ± 0.20	1.15 ± 0.35
AA	34	5.42 ± 1.02	2.51 (2.06)	1.35 ± 0.37	2.99 ± 0.47	1.15 ± 0.16	1.17 ± 0.26	1.04 ± 0.23
*F*		2.043	6.362	2.868	3.980	4.102	0.259	1.423
*P*		0.131	0.042	0.058	0.019	0.017	0.772	0.242
Non‐hypertriglyceridaemia								
GG	924	4.85 ± 0.87	1.18 (0.99)	1.88 ± 0.41	2.76 ± 0.43	1.33 ± 0.22	1.00 ± 0.24	1.40 ± 0.37
AG	370	4.90 ± 0.81	1.24 (1.03)	1.84 ± 0.50	2.78 ± 0.34	1.32 ± 0.20	1.01 ± 0.23	1.37 ± 0.40
AA	35	5.02 ± 0.74	1.40 (1.19)	1.57 ± 0.28	2.80 ± 0.39	1.17 ± 0.13	1.04 ± 0.16	1.16 ± 0.29
*F*		0.710	30.258	5.766	0.398	11.022	0.180	5.846
*P*		0.492	0.000	0.003	0.672	0.000	0.835	0.003
*GALNT2* rs2760537 C>T								
Hypertriglyceridaemia								
CC	215	5.23 ± 0.95	2.09 (1.84)	1.65 ± 0.60	2.87 ± 0.49	1.28 ± 0.23	1.14 ± 0.27	1.18 ± 0.39
CT	236	5.29 ± 0.88	2.17 (1.90)	1.57 ± 0.40	2.99 ± 0.40	1.27 ± 0.20	1.14 ± 0.24	1.17 ± 0.35
TT	89	5.32 ± 0.94	2.50 (1.94)	1.55 ± 0.40	3.01 ± 0.45	1.27 ± 0.20	1.16 ± 0.23	1.13 ± 0.30
*F*		0.206	8.583	1.722	4.037	0.565	0.457	0.518
*P*		0.814	0.014	0.180	0.018	0.569	0.634	0.596
Non‐hypertriglyceridaemia								
CC	558	4.78 ± 0.83	1.17 (0.97)	1.88 ± 0.57	2.74 ± 0.41	1.33 ± 0.21	0.99 ± 0.22	1.39 ± 0.35
CT	614	4.92 ± 0.84	1.21 (1.02)	1.87 ± 0.44	2.79 ± 0.39	1.33 ± 0.20	1.01 ± 0.24	1.38 ± 0.40
TT	157	4.98 ± 0.93	1.25 (1.09)	1.81 ± 0.48	2.83 ± 0.49	1.32 ± 0.22	1.03 ± 0.28	1.38 ± 0.43
*F*		5.129	17.473	2.078	2.793	0.330	1.499	0.255
*P*		0.006	0.000	0.126	0.062	0.719	0.224	0.775
*GALNT2* rs4846913 C>A								
Hypertriglyceridaemia								
CC	348	5.05 ± 1.06	2.12 (1.87)	1.63 ± 0.51	2.76 ± 0.70	1.30 ± 0.23	1.13 ± 0.24	1.19 ± 0.37
AC	166	5.28 ± 0.93	2.16 (1.88)	1.56 ± 0.48	2.98 ± 0.43	1.23 ± 0.18	1.15 ± 0.25	1.10 ± 0.26
AA	26	5.34 ± 0.87	2.62 (2.09)	1.47 ± 0.32	3.02 ± 0.40	1.19 ± 0.21	1.16 ± 0.21	1.10 ± 0.30
*F*		1.175	7.314	2.303	3.290	6.814	0.180	4.669
*P*		0.310	0.026	0.101	0.038	0.001	0.835	0.010
Non‐hypertriglyceridaemia								
CC	964	4.80 ± 0.78	1.17 (0.99)	1.87 ± 0.49	2.74 ± 0.40	1.34 ± 0.22	1.00 ± 0.23	1.40 ± 0.38
AC	325	4.87 ± 0.87	1.24 (1.06)	1.76 ± 0.44	2.78 ± 0.42	1.29 ± 0.19	1.01 ± 0.24	1.36 ± 0.37
AA	40	5.27 ± 0.83	1.51 (1.37)	1.76 ± 0.45	2.90 ± 0.38	1.22 ± 0.21	1.06 ± 0.20	1.18 ± 0.30
*F*		4.810	66.540	6.626	2.481	10.045	0.606	3.704
*P*		0.008	0.000	0.001	0.084	0.000	0.546	0.025
*GALNT2* rs11122316G>A								
Hypertriglyceridaemia								
GG	179	5.27 ± 0.88	2.05 (1.83)	1.63 ± 0.43	2.96 ± 0.46	1.27 ± 0.24	1.14 ± 0.24	1.17 ± 0.37
AG	279	5.29 ± 0.99	2.15 (1.87)	1.60 ± 0.54	2.99 ± 0.42	1.27 ± 0.21	1.15 ± 0.24	1.16 ± 0.32
AA	82	5.37 ± 0.89	2.30 (1.92)	1.59 ± 0.46	3.03 ± 0.38	1.27 ± 0.21	1.17 ± 0.22	1.13 ± 0.32
*F*		0.352	10.413	0.011	1.016	0.110	0.438	0.432
*P*		0.703	0.005	0.990	0.363	0.896	0.645	0.650
Non‐hypertriglyceridaemia								
GG	551	4.81 ± 0.85	1.15 (0.97)	1.88 ± 0.52	2.75 ± 0.40	1.34 ± 0.20	0.99 ± 0.22	1.41 ± 0.35
AG	618	4.84 ± 0.80	1.23 (1.02)	1.86 ± 0.48	2.79 ± 0.43	1.34 ± 0.20	1.00 ± 0.22	1.39 ± 0.38
AA	160	4.94 ± 0.87	1.26 (1.15)	1.82 ± 0.46	2.80 ± 0.39	1.31 ± 0.23	1.02 ± 0.25	1.37 ± 0.38
*F*		2.868	24.754	1.702	1.606	2.803	0.893	0.463
*P*		0.057	0.000	0.183	0.201	0.061	0.410	0.629

The values of TG were presented as median (interquartile range). The difference among the genotypes was determined by the Kruskal–Wallis test or the Wilcoxon‐Mann–Whitney test.

TC: total cholesterol; TG: triglyceride; HDL‐C: high‐density lipoprotein cholesterol; LDL‐C: low‐density lipoprotein cholesterol; ApoA1: apolipoprotein A1; ApoB: apolipoprotein B; ApoA1/A poB: the ratio of a polipoprote in A1 to apolipoprote in B.

After adjusting age, gender, BMI, smoking and alcohol consumption, logistic regression analysis showed that the SNPs of rs10889332, rs615563, rs7552841, rs2760537 and rs4846913 were associated with HCH (*P* < 0.05). The SNPs of rs615563, rs7552841, rs11206517, rs1997947, rs4846913 and rs11122316 were associated with HTG (*P* < 0.05; Tables 3 and 4).

### Haplotypes and the risk of hyperlipidaemia

As shown in Table 7, the haplotype of G‐C‐G‐C‐T‐G‐C‐C‐G [in the order of *DOCK7* rs1168013 (G>C), rs10889332 (C>T); *PCSK9* rs615563 (G>A), rs7552841 (C>T), rs11206517 (T>G); and *GALNT2* rs1997947 (G>A), rs2760537 (C>T), rs4846913 (C>A) and rs11122316 (G>A) SNPs] was the most common haplotype and represented >10% of the samples. The haplotype of C‐C‐G‐C‐T‐G‐C‐C‐G was associated with increased risk of HCH (OR: 3.29, 95% CI: 1.81, 6.00, *P* < 0.001) and HTG (OR: 3.99, 95% CI: 1.81, 8.77, *P* < 0.001). The haplotypes of G‐C‐G‐C‐T‐G‐C‐C‐A and G‐C‐G‐C‐T‐G‐T‐C‐G were associated with increased risk of HCH (OR: 1.68, 95% CI: 1.15, 2.46, *P* = 0.007 and OR: 1.67, 95% CI: 1.13, 2.47, *P* = 0.009 respectively). The haplotypes of C‐T‐G‐C‐T‐G‐C‐C‐G (OR: 2.02, 95% CI: 1.20, 3.41, *P* = 0.007), G‐C‐A‐C‐T‐G‐C‐C‐G (OR: 1.66, 95% CI: 1.00, 2.75, *P* = 0.046) and G‐C‐G‐C‐T‐G‐C‐C‐A (OR: 3.38, 95% CI: 2.13, 5.36, *P* < 0.001) were associated with an increased risk of HTG. The haplotypes of C‐C‐G‐C‐T‐G‐C‐C‐A and G‐C‐G‐T‐T‐G‐T‐C‐G were associated with a reduced risk of HCH (OR: 0.23, 95% CI: 0.14, 0.37, *P* < 0.001 and OR: 0.51, 95% CI: 0.30, 0.86, *P* = 0.010 respectively) and HTG (OR: 0.36, 95% CI: 0.23, 0.56, *P* < 0.001 and OR: 0.10, 95% CI: 0.05, 0.17, *P* < 0.001 respectively). The haplotypes of G‐C‐G‐C‐T‐G‐T‐C‐A and G‐C‐G‐T‐T‐G‐T‐C‐G were associated with a reduced risk of HTG (OR: 0.40, 95% CI: 0.27, 0.58, *P* < 0.001 and OR: 0.10, 95% CI: 0.05, 0.17, *P* < 0.001 respectively).

**Table 7:** The association between the DOCK7, PCSK9 and GALNT2 haplotypes and hypercholesterolaemia/hypertriglyceridaemia

Haplotypes	Hypercholesterolaemia				Hypertriglyceridaemia			
	Cases, *n* (feq)	Control, *n* (feq)	OR (95% CI)	*P*‐value	Cases, *n* (feq)	Control, *n* (feq)	OR (95% CI)	*P*‐value
C‐C‐G‐C‐T‐G‐C‐C‐A	26 (0.011)	49 (0.033)	0.23 (0.14, 0.37)	2.01 × 10^−10^	46 (0.017)	37 (0.034)	0.36 (0.23, 0.56)	3.67 × 10^−6^
C‐C‐G‐C‐T‐G‐C‐C‐G	83 (0.037)	13 (0.009)	3.29 (1.81, 6.00)	4.12 × 10^−5^	85 (0.032)	7 (0.006)	3.99 (1.81, 8.77)	0.000
C‐T‐G‐C‐T‐G‐C‐C‐G	75 (0.033)	35 (0.024)	1.01 (0.67, 1.54)	0.949	110 (0.041)	17 (0.016)	2.02 (1.20, 3.41)	0.007
G‐C‐A‐C‐T‐G‐C‐C‐G	61 (0.027)	44 (0.030)	0.89 (0.60, 1.32)	0.572	102 (0.038)	19 (0.018)	1.66 (1.00, 2.75)	0.046
G‐C‐G‐C‐T‐G‐C‐C‐A	130 (0.057)	39 (0.027)	1.68 (1.15, 2.46)	0.007	210 (0.079)	22 (0.020)	3.38 (2.13, 5.36)	5.02 × 10^−8^
G‐C‐G‐C‐T‐G‐C‐C‐G	359 (0.158)	189 (0.129)	0.83 (0.66, 1.05)	0.118	360 (0.135)	116 (0.107)	0.92 (0.71, 1.18)	0.496
G‐C‐G‐C‐T‐G‐T‐C‐A	85 (0.037)	55 (0.038)	0.70 (0.49, 1.00)	0.051	73 (0.027)	51 (0.047)	0.40 (0.27, 0.58)	1.05 × 10^−6^
G‐C‐G‐C‐T‐G‐T‐C‐G	122 (0.054)	37 (0.025)	1.67 (1.13, 2.47)	0.009	158 (0.060)	41 (0.038)	1.21 (0.83, 1.74)	0.320
G‐C‐G‐T‐T‐G‐T‐C‐G	26 (0.011)	32 (0.022)	0.51 (0.30, 0.86)	0.010	15 (0.006)	42 (0.039)	0.10 (0.05, 0.17)	2.14 × 10^−20^

The haplotypes were composed in the order of *DOCK7* rs1168013 (G>C), *DOCK7* rs10889332 (C>T), *PCSK9* rs615563 (G>A), *PCSK9* rs7552841 (C>T), *PCSK9* rs11206517 (T>G), *GALNT2* rs1997947 (G>A), *GALNT2* rs2760537 (C>T), *GALNT2* rs4846913 (C>A) and *GALNT2* rs11122316 (G>A) SNPs.

### Gene–Gene interaction for hyperlipidaemia

Table 8 shows the impacts of combination among the *DOCK7, PCSK9* and *GALNT2* SNPs, which were analysed by GMDR. The two‐ and three‐locus models showed a significant association with the risk of HCH and HTG (*P* < 0.01–0.001). The two‐locus model was chosen as the best one, owing to the fact of having the highest level of testing accuracy (54.71%) for HCH and good cross‐validation consistency (7/10).The three‐locus model was chosen as the best one, owing to the fact of having the highest level of testing accuracy (59.00% for HTG) and good cross‐validation consistency (9/10).

**Table 8:** Best inter‐locus interaction models identified by the generalized multifactor dimensionality reduction method

Locus no	Best combination for HTC	Cross‐validation consistency	Testing accuracy	*P*‐value
2	rs10889332‐ rs1997947	7/10	0.5471	0.0107
3	rs1168013‐ rs7552841‐ rs1997947	2/10	0.5130	0.1719

Locus no	Best combination for HTG	Cross‐validation consistency	Testing accuracy	*P*‐value
2	rs615563‐ rs7552841	9/10	0.5814	0.0010
3	rs615563‐rs7552841‐ rs4847913	9/10	0.5900	0.0107

HTC: hypercholesterolaemia; HTG: hypertriglyceridaemia.

## Discussion

The main findings of this study encompass (*i*) the associations of the *DOCK7*,* PCSK9* and *GALNT2* SNPs with serum lipid levels in individuals with HCH and HTG; (*ii*) the correlation of their haplotypes with HCH/HTG and (*iii*) possible gene–gene interaction among these variants to influence HCH/HTG. This is the first report on the inter‐locus interaction among the *DOCK7*,* PCSK9* and *GALNT2* SNPs on serum lipid levels. The observed allele frequencies of the remaining nine SNPs in the non‐HCH/non‐HTG populations were consistent with those of the International Hapmap Chinese Han Beijing sample (http://hapmap.ncbi.nlm.nih.gov/cgi-perl/gbrowse/hapmap27_B36/).

Recently, a couple of previous reports found that the individuals with transferability and fine mapping of genome‐wide‐associated loci, *DOCK7* rs2131925‐T‐allele, was associated with serum TC levels in African‐Americans 44, genetic loci rs10889353‐C‐allele was correlation with TC and TG levels in the Chinese population 45, and rs636523‐T‐allele near *DOCK7* was related to plasma TG levels in the Jackson Heart Study 25. Likewise, in some population's large‐scale association studies, the *PCSK9* rs17111557‐T‐allele carriers had lower HDL‐C than the C‐allele carriers in Brazilians 46, common variants of rs12067569 and rs505151 in *PCSK9* were significantly associated with higher LDL‐C and for rare variants rs11591147 (R46L, MAF = 0.9%) was associated with lower LDL‐C in American‐Indians 38, and the E670G SNP in the *PCSK9* was associated with polygenic HCH in men, but not in women 47. Moreover, in a large‐scale GWAS, the *GALNT2* variants were associated with quantitative change in serum lipid levels. In particular, *GALNT2*G allele frequency of rs4846914 showed correlation with TG levels in the Korean populations 48 and no correlation with TG levels in healthy Roma and Hungarian populations 49, segregation of *GALNT2* D314A mutations in Caucasian families with extremely high HDL‐C 50, and heterozygosity for a loss‐of‐function mutation in *GALNT2* improves plasma TG clearance in man 51. In the present study, we found that the alleles of rs10889332‐T, rs615563‐A, rs7552841‐T, rs1997947‐A, rs2760537‐T and rs4846913‐A were more frequent in HCH/HTG than in non‐HCH/non‐HTG populations. The alleles of rs11206517‐G and rs11122316‐A were more frequent just in HTG than in non‐HTG populations. The levels of TC (rs10889332 and rs7552841), TG (rs10889332, rs7552841, rs11206517, rs1997947, rs4846913 and rs11122316), HDL‐C (rs1168013, rs11206517, rs1997947 and rs4846913), LDL‐C (rs7552841 and rs1997947), ApoA1 (rs10889332, rs1997947 and rs4846913), ApoB (rs1168013, rs10889332 and rs7552841) and the ratio of ApoA1 to ApoB (rs1168013, rs10889332 and rs7552841) in the hypercholesterolaemic participants were different between the three genotypes (*P* < 0.005–0.001), whereas the levels of TC (rs1997947 and rs2760537), TG (rs10889332, rs615563, rs7552841, rs1997947, rs4846913 and rs11122316), ApoB (rs615563, rs7552841 and rs1997947) and the ratio of ApoA1 to ApoB (rs4846913) in the normocholesterolaemic individuals were different between the three genotypes. Likewise, the levels of TG (rs1168013, rs10889332 and rs7552841), ApoA1 (rs4846913) and the ratio of ApoA1 to ApoB (rs10889332) in the hypertriglyceridaemic population were different between the genotypes, whereas the levels of TC (rs1088933, rs615563 and rs7552841), TG (rs10889332, rs615563, rs1997947, rs2760537, rs4846913 and rs11122316) and HDL‐C (rs1168013, rs615563, rs11206517, rs1997947 and rs4846913), LDL‐C (rs10889332 and rs7552841), ApoA1 (rs1997947 and rs4846913), ApoB (rs10889332, rs615563, rs7552841 and rs11206517) and the ratio of ApoA1 to ApoB (rs615563, rs7552841, rs11206517 and rs1997947) in the normotriglyceridaemic population were different between the genotypes. The reason for these discrepancies among the studies is not fully understood. The differences in the genetic background, linkage disequilibrium pattern and/or environmental factors may partly explain these discrepancies.

Alirocumab, an inhibitor of PCSK9, significantly reduced levels of LDL‐C when added to statin therapy administered at the maximum tolerated dose 52. Current guidelines suggest high‐intensity statin treatment for most high‐risk patients 53. However, only 47% of the study patients were receiving high‐dose statins, resulting in a mean baseline LDL‐C level of 122 mg/dl. Treatment with high‐dose statins would have brought a much higher percentage of patients in the placebo group to the goal of an LDL‐C level of less than 70 mg/dl 54. In addition, appropriate use of high‐dose statins would have been associated with a lower rate of major adverse cardiovascular events in the placebo group 55, 56. Thus, a strategy of not exploiting the maximum potential of statins in high‐risk patients may have overestimated the benefit of PCSK9 inhibition. The efficacy and safety of the PCSK9 inhibitor, alirocumab, in reducing lipids and cardiovascular events may be influenced by these above SNPs. It is expected that the association of genetic susceptibility of PCSK9 polymorphisms and the lipid‐lowering efficacy of alirocumab treatment in the levels of LDL‐C will be elucidated in a not too distant future. What is more, the participants with a history of taking lipid‐modulating medications such as statins, fibrates or PCSK9 inhibitors were excluded in present study. But, the associations between the above genes and serum lipid levels and lipid‐lowering efficacy of treatment are also needed to further explore, especially, when using LDL‐C and TG levels to divide groups.

When assessing the association of the *DOCK7*,* PCSK9* and *GALNT2* SNPs and the risk of hyperlipidaemia, this study showed that although the variants of *DOCK7* rs1168013, *PCSK9* rs2760537 and *GALNT2* rs11122316 did not reach statistically significant association with HCH/HTG risk, they, in moderation with other SNPs, achieved significant association with the risk of HCH/HTG. In addition, we noticed that the haplotype of C‐C‐G‐C‐T‐G‐C‐C‐G, carrying rs11122316‐G‐allele, was associated with an increased risk of HCH and HTG. The haplotypes of C‐C‐G‐C‐T‐G‐C‐C‐A and G‐C‐G‐T‐T‐G‐T‐C‐G were associated with reduced risk of HCH and HTG. The haplotypes of G‐C‐G‐C‐T‐G‐C‐C‐A and G‐C‐G‐C‐T‐G‐T‐C‐G were associated with an increased risk of HCH. The haplotypes of C‐T‐G‐C‐T‐G‐C‐C‐G, G‐C‐A‐C‐T‐G‐C‐C‐G and G‐C‐G‐C‐T‐G‐C‐C‐A were associated with an increased risk of HTG. The haplotypes of G‐C‐G‐C‐T‐G‐T‐C‐A and G‐C‐G‐T‐T‐G‐T‐C‐G were associated with a reduced risk of HTG.

On GMDR analysis, an inter‐locus interaction among the *DOCK7*,* PCSK9* and *GALNT2* SNPs on serum lipid levels was found in this study. The interactions of rs10889332–rs1997947 were associated with the risk of HCH, and rs615563–rs7552841, and/or rs615563–rs7552841–rs4847913 were associated with the risk of HTG. In multi‐locus (GMDR) analyses, a significant association with HCH and HTG was found in two‐ to three‐locus models. These findings indicate that a potential gene–gene interaction might exist among the *DOCK7*,* PCSK9* and *GALNT2* SNPs. Unfortunately, no previous study has investigated the inter‐locus interaction between these SNPs, and therefore we cannot make comparisons with our results. Although, a statistically significant SNP–SNP interaction was noted in this study, the biological mechanism underlying these genes and their interactions is still yet to be defined.

### Study limitations

There are several potential limitations in our study. First, the number of participants available for MAF of some SNPs was not high enough to calculate a strong power as compared with many previous GWAS and replication studies. Hence, further studies with larger sample size are needed to confirm our results. Second, we were unable to alleviate the effect of diet during the statistical analysis. Third, although we have detected the interactions of the *DOCK7*,* PCSK9* and *GALNT2* SNPs on hyperlipidaemia in this study, many unmeasured environmental and genetic factors still need to be considered. Besides, the interactions of gene–environment and environment–environment on serum lipid levels remain to be determined. For the clear understanding of biological mechanism underlying hyperlipidaemia, an enormous amount of common variants with small effects and rare variants with large effects still remain to be determined. What is more, the relevance of this finding has to be defined in further high calibre of studies including incorporating the genetic information of the *DOCK7, PCSK9* and *GALNT2* SNPs and their haplotypes and *in vitro* functional studies to confirm the impact of a variant on a molecular level.

## Conclusions

Our study confirmed that the genetic variants are replicable in the Southern Chinese hyperlipidaemic and normolipidaemic populations. The haplotype of C‐C‐G‐C‐T‐G‐C‐C‐G was associated with an increased risk of HCH and HTG. The haplotypes of C‐C‐G‐C‐T‐G‐C‐C‐A and G‐C‐G‐T‐T‐G‐T‐C‐G were associated with a reduced risk of HCH and HTG. The haplotypes of G‐C‐G‐C‐T‐G‐C‐C‐A and G‐C‐G‐C‐T‐G‐T‐C‐G were associated with an increased risk of HCH. The haplotypes of C‐T‐G‐C‐T‐G‐C‐C‐G, G‐C‐A‐C‐T‐G‐C‐C‐G and G‐C‐G‐C‐T‐G‐C‐C‐A were associated with an increased risk of HTG. The haplotypes of G‐C‐G‐C‐T‐G‐T‐C‐A and G‐C‐G‐T‐T‐G‐T‐C‐G were associated with a reduced risk of HTG. In addition, possible inter‐locus interactions among the *DOCK7*,* PCSK9* and *GALNT2* SNPs are also noted. However, further functional studies of these genes are still required to clarify which SNPs are functional and how these genes actually affect the serum lipid levels.

Taken all of facts into consideration, it is possible that the significant SNPs identified in the *DOCK7*,* PCSK9* and *GALNT2* region might be in high linkage disequilibrium with some of the functional SNPs in other genes, which is known to affect the lipid metabolism. Thus, an in‐depth study of the biological actions of these genes is crucial to clarify which SNPs are functional and how these genes actually affect the serum lipid levels. It is expected that the physiological function of *DOCK7*,* PCSK9* and *GALNT2* will be elucidated in a not too distant future.

## Conflicts of interest

The authors confirm that there are no conflicts of interest.
